# Facial soft-tissue analysis in undergraduate dental education: comparison of 2D photographs and images derived from 3D facial scans

**DOI:** 10.1186/s12909-026-08968-4

**Published:** 2026-03-12

**Authors:** Janine Sambale, Albrecht von Bülow, Anahita Jablonski-Momeni, Heike Korbmacher-Steiner

**Affiliations:** Department of Orthodontics, Clinic of Dentistry, Marburg University, Georg-Voigt-Str. 3, Marburg, 35039 Germany

**Keywords:** Facial soft-tissue analysis, Three-dimensional facial scan, 3D imaging, Undergraduate dental education, Curricula, Digitalization

## Abstract

**Background:**

Two-dimensional (2D) photographs are routinely used for teaching facial soft-tissue analysis, whereas three-dimensional (3D) facial scans are increasingly used in clinical orthodontics. This prospective educational study evaluated diagnostic agreement when dental students assessed standardized orthodontic soft-tissue parameters using 2D photographs and 3D facial scan-derived images.

**Methods:**

93 dental students (7th, 9th, and 10th semesters) performed standardized soft-tissue analyses using 2D photographs, 3D color facial scans, and 3D grayscale facial scans. Paired images from 20 orthodontic patients were presented in standardized printed formats. Assessments were conducted sequentially at three time points one week apart (2D, 3D color, 3D grayscale). A consensus reference standard established by orthodontic experts served as the gold standard. Agreement was assessed using Cohen’s kappa.

**Results:**

67 students completed the 2D assessment, and 50 students completed each 3D assessment. Agreement for objective parameters was highest for 2D photographs and lowest for 3D grayscale images. Compared with 2D images, agreement decreased for most objective parameters with 3D color scans and further decreased for 3D grayscale images. Facial thirds showed a stepwise reduction in agreement across modalities. Profile classification according to A.M. Schwarz showed the highest agreement with 3D color scans. Higher-semester students achieved greater agreement for most objective parameters, whereas agreement for subjective variables was highest for 2D images.

**Conclusion:**

Standardized 2D orthodontic soft-tissue analysis can be transferred to facial scan-derived images with limitations. Color facial scans provide more reliable diagnostic conditions than grayscale scans, and diagnostic agreement improves with educational experience, supporting integration of 3D imaging into undergraduate orthodontic training.

## Introduction

Traditionally, facial diagnostics relies on standardized extraoral frontal and lateral photographs, which are indispensable for treatment planning, growth assessment, and longitudinal evaluation of therapeutic outcomes. Facial soft-tissue analysis is a fundamental component of orthodontic and maxillofacial diagnostics because functional harmonization, profile harmony and facial esthetics are central to treatment objectives and patient satisfaction. Such analyses have traditionally been performed using two-dimensional (2D) photographs taken with digital single-lens reflex cameras [1].

Standard orthodontic photographic analysis includes frontal and lateral views that provide complementary diagnostic information, with frontal images assessing symmetry and proportions, and lateral views evaluating sagittal facial relationships.

In recent years, however, three-dimensional (3D) facial scanning technologies have become increasingly integrated into dental and orthodontic diagnostics [2–5].

These systems capture facial morphology together with surface texture, allowing virtual models to be visualized from any perspective and digitally aligned with other diagnostic records, such as intraoral scans, lateral cephalograms, or cone-beam computed tomography datasets [4, 6, 7]. Beyond their diagnostic potential, 3D facial scans offer advantages for patient communication, interdisciplinary case discussions, and visualization of treatment planning, emphasizing their growing relevance in both clinical practice and dental education.

With the introduction of the new German Dental Licensing Regulations in the year 2021, the integration of digital diagnostic technologies, including 3D facial scans, has gained increasing importance in undergraduate dental curricula [8].

This study aimed to assess the performance of undergraduate dental students when conducting standardized facial soft-tissue analysis using 2D photographs, 3D color facial scans, and 3D grayscale facial scans. The primary focus was on objectively measurable parameters, while subjective soft-tissue characteristics were evaluated as a secondary outcome. It was hypothesized that diagnostic performance would differ between imaging modalities, with greater variability for 3D images compared with 2D photographs, and that diagnostic accuracy would increase with advancing semester level.

## Materials and methods

This prospective study was conducted in accordance with the Declaration of Helsinki and was approved by the Ethics Committee of the University of Marburg (reg. no. 24–337 BO). Written informed consent was obtained from all patients and, where applicable, their legal guardians for the acquisition and use of facial images. The students provided written informed consent to take part in the study and to perform the image-based assessments.

A priori sample size calculation (G*Power 3.1 [9]) indicated that a total sample of 72 participants (3 experts and 69 students) was required to detect a large effect size (d = 1.4) with 80% power at a 5% significance level.

### Inclusion criteria for patients

Patients were eligible for inclusion if they met the following criteria:

- availability of complete sets of 2D extraoral photographs and 3D facial scans acquired simultaneously,
- age within the typical range for orthodontic treatment (9–16 years),
- provision of written informed consent by both the patient and their legal guardians.

### Inclusion criteria for students

Students were eligible to participate if they:

- were enrolled in the 7th, 9th, or 10th semester of dental school during the winter term 2024/2025,
- participated voluntarily and provided written informed consent.

### Data collection

**Fig. 1:**
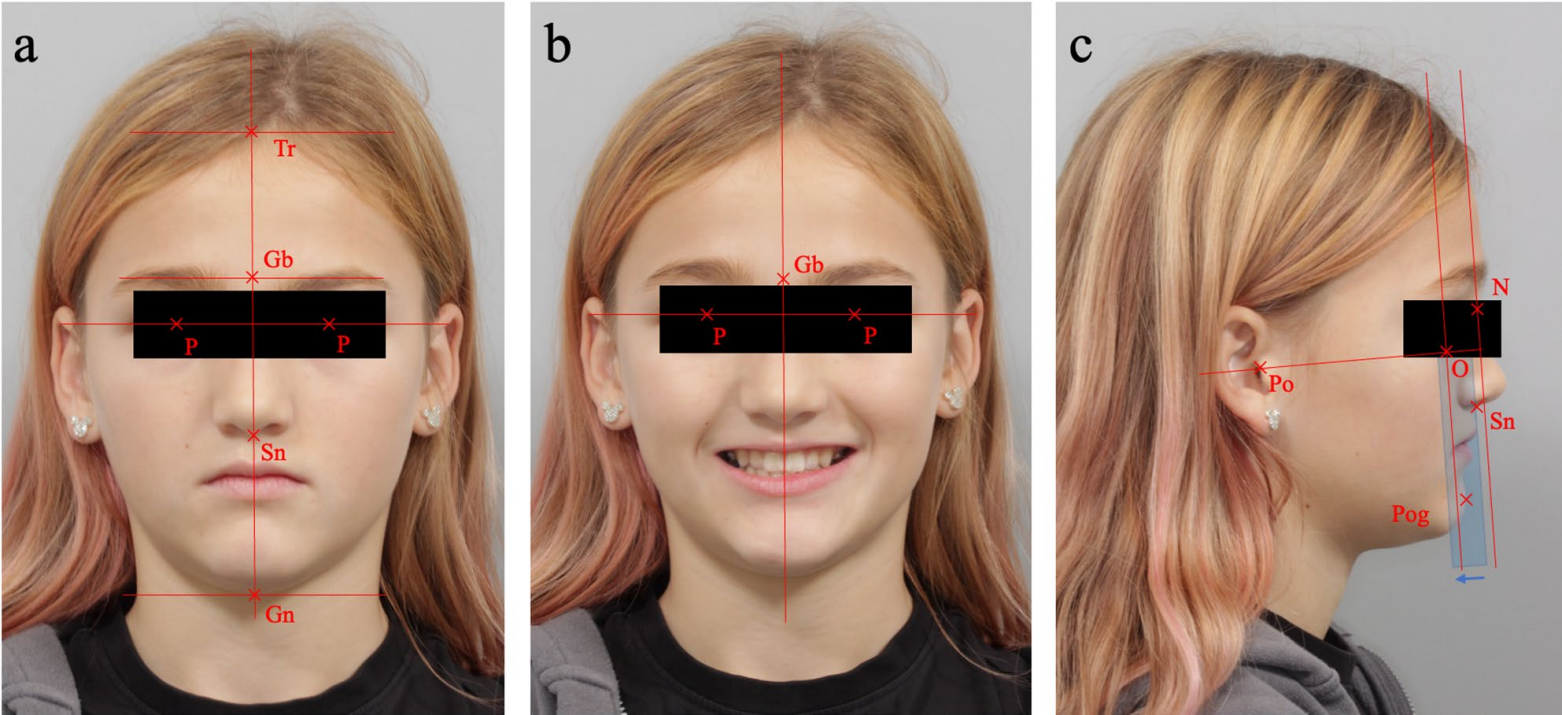
Representative example of two-dimensional (2D) images used for orthodontic soft-tissue analysis: (**a**) frontal view at rest (enface), (**b**) frontal view during smiling (en face smiling), (**c**) lateral profile view (profile): the blue rectangle illustrates the parallel displacement of the jaw profile field. Abbr.: Tr, trichion; Gb, glabella; Gn, gnathion; N, nasion; Or, orbitale; P, pupil; Po, porion; Pog, pogonion; Sn, subnasale

**Fig. 2:**
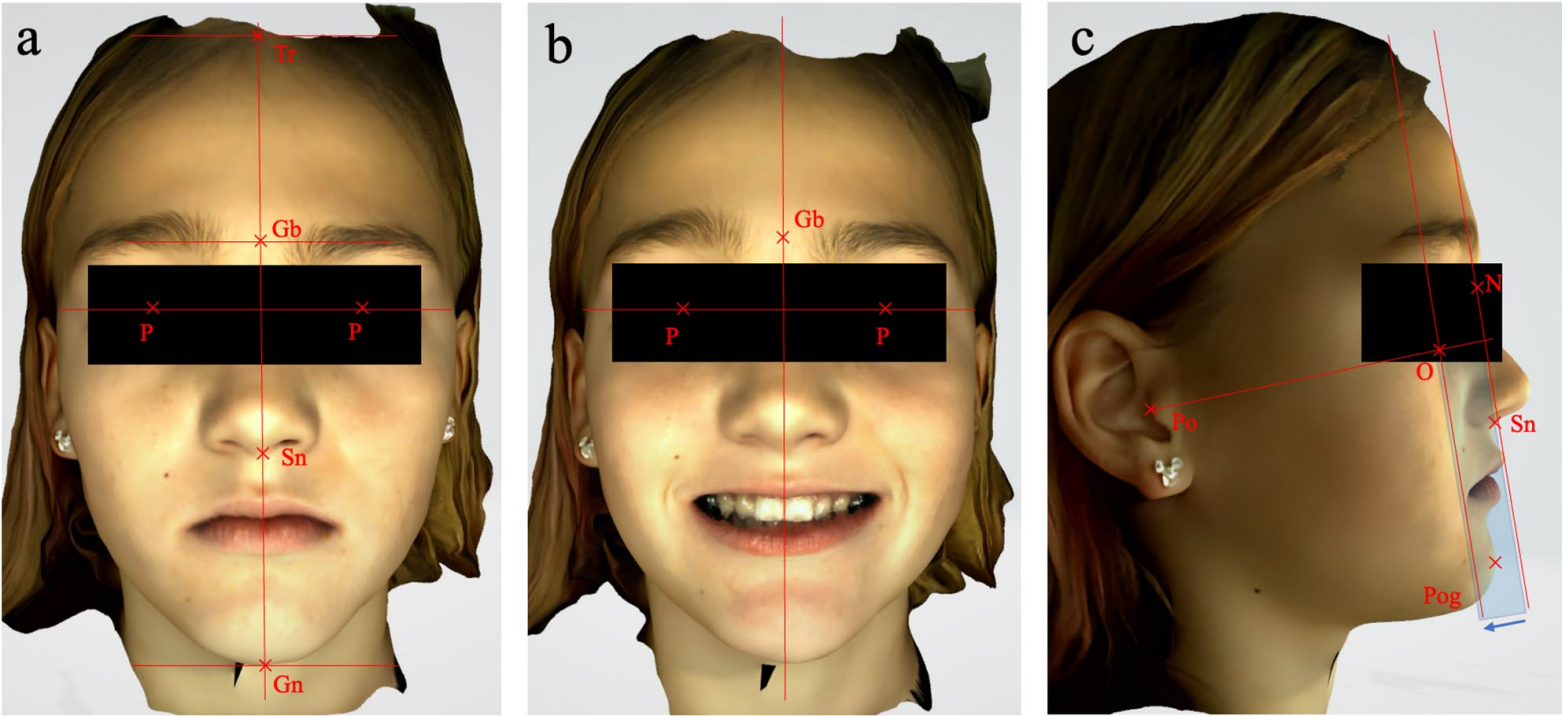
Representative example of the same patient of three-dimensional (3D) colour images used for orthodontic soft-tissue analysis: (**a**) frontal view at rest (enface), (**b**) frontal view during smiling (en face smiling), (**c**) lateral profile view (profile): the blue rectangle illustrates the parallel displacement of the jaw profile field. Abbr.: Tr, trichion; Gb, glabella; Gn, gnathion; N, nasion; Or, orbitale; P, pupil; Po, porion; Pog, pogonion; Sn, subnasale

**Fig. 3:**
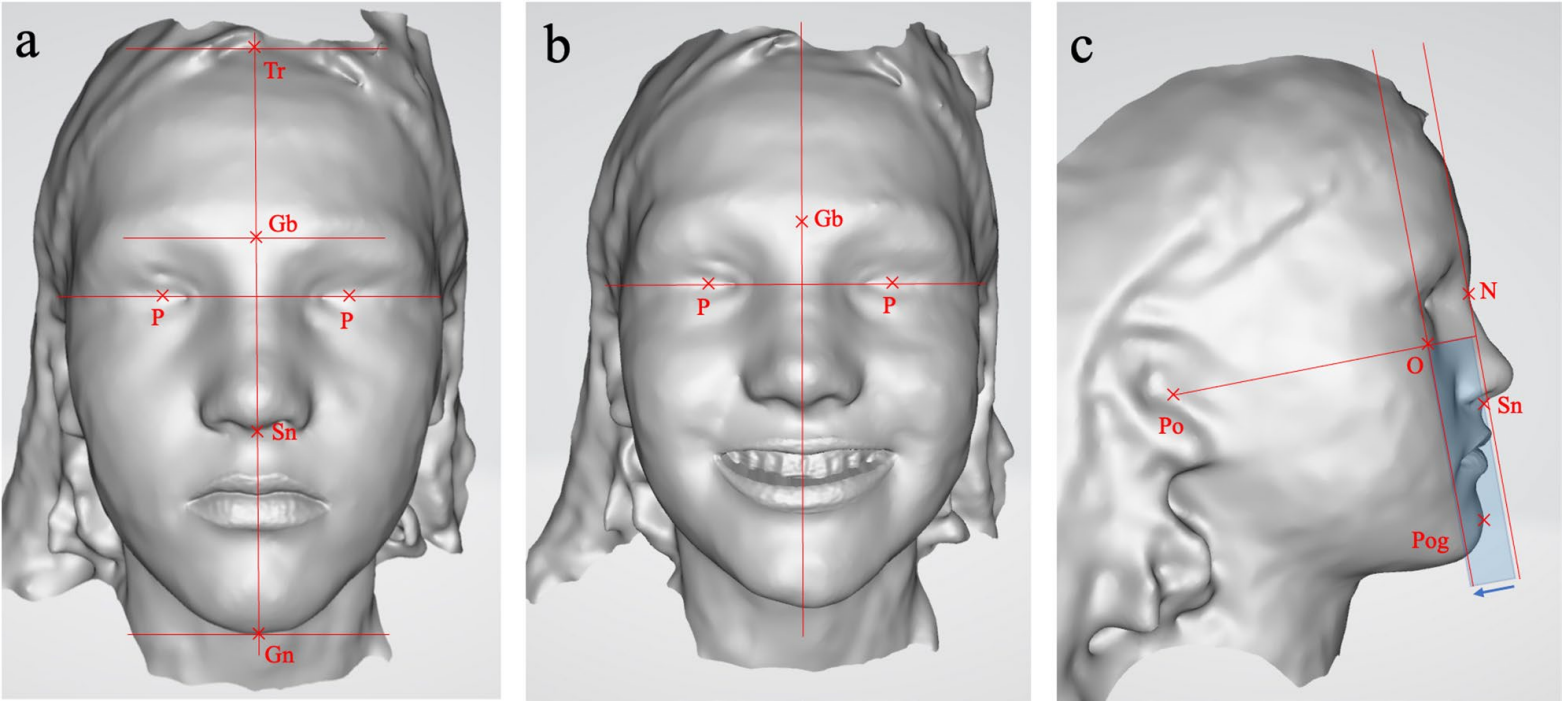
Representative example of the same patient of three-dimensional (3D) grayscale images used for orthodontic soft-tissue analysis: (**a**) frontal view at rest (enface), (**b**) frontal view during smiling (en face smiling), (**c**) lateral profile view (profile): the blue rectangle illustrates the parallel displacement of the jaw profile field. Abbr.: Tr, trichion; Gb, glabella; Gn, gnathion; N, nasion; Or, orbitale; P, pupil; Po, porion; Pog, pogonion; Sn, subnasale

Two-dimensional (2D) extraoral photographs and three-dimensional (3D) facial scans were obtained from 20 patients, aged 6 to 16 years, who presented to the orthodontic department at the University of Marburg between December 2024 and January 2025. Photographs were taken with a Canon EOS 650D single-lens reflex camera in an upright seated position, following a standardized protocol. Three standardized views were acquired from the same patient: a frontal view (en face), a frontal view with a relaxed smile (en face smiling), and a lateral profile view (profile). All landmarks and reference lines are presented in Table 1 and illustrated in Figs. 1, 2 and 3.

**Table 1:** Landmarks and reference lines: abbreviations and definitions used for facial soft-tissue analysis

Landmark	Abbreviation	Definition
Trichion	Tr	Point at which the mid-sagittal line intersects the anterior hairline of the forehead.
Glabella	Gb	Most prominent midline point between the eyebrows on the frontal bone.
Nasion	N	Most posterior depression at the nasal root located between the forehead and the nose.
Pupil center	P	Center of the visible pupil, used to define the bipupillary line.
Orbitale	O	Lowest point on the infraorbital margin, located approximately one eyelid width below the lower eyelid.
Porion	Po	Superior point of the external acoustic meatus.
Subnasale	Sn	Midline point at the junction between the columella base and the upper lip.
Pogonion	Pg	Most anterior point of the soft-tissue contour of the chin.
Gnathion	Gn	Most inferior point on the soft-tissue contour of the chin in the mid-sagittal plane.
Reference line		
Bipupillary line	-	Horizontal line connecting the centers of both pupils, serving as the transverse reference for en face analysis.
Mid-sagittal line	-	Vertical line perpendicular to the bipupillary line intersecting it at its midpoint, serving as the reference for facial symmetry and assessment of chin and dental midline deviations.
Upper facial third	-	Vertical distance between trichion and glabella measured along the mid-sagittal line.
Middle facial third	-	Vertical distance between glabella and subnasale measured along the mid-sagittal line.
Lower facial third	-	Vertical distance between subnasale and gnathion measured along the mid-sagittal plane.
Horizontal reference line	-	Horizontal line defined by porion and orbitale (Po - O), serving as the reference line for profile analysis.
Anterior perpendicular line	-	Vertical line perpendicular to the horizontal reference line passing through nasion.
Posterior perpendicular line	-	Vertical line perpendicular to the horizontal reference line intersecting orbitale and the horizontal reference line.
Facial profile type (A. M. Schwarz)	-	Classification of sagittal facial profile based on the relative positions of subnasale and pogonion with respect to the perpendicular reference lines, defining nine distinct profile types.
Nasolabial angle	-	Angle formed between the columella and the philtrum, reflecting nasal projection and upper lip inclination.

Facial scans were captured using the RayFace 200 system (Ray Europe GmbH, Eschborn, Germany) in an upright seated position as well. Patients were instructed to look straight at the central mirror of the device and focus on their reflection’s eyes. In this position, one scan was acquired with relaxed lips and another with smiling lips.

The RayFace 200 is a stationary facial scanning system that uses stripe projection technology. It records a facial scan in 0.5 s using 13 cameras and a digital light processing (DLP) projector. The software then reconstructs the images into a 3D facial model. From each of the two facial scans, colored (Fig. 2) and grayscale images (Fig. 3) corresponding to the three photograph views were exported and printed. The grayscale images are based on facial scans from which surface texture information has been removed.

### Assessment form

The assessment form (Appendix I) included the standardized landmarks and assessment criteria commonly used in orthodontic facial soft-tissue analysis. All variables were applied identically to the printed 2D photographs and to the printed 3D facial scans. In total, the assessment form consisted of 13 assessment items, comprising 6 objective (measurable) variables and 7 subjective (qualitative) variables.

Objective (measurable) variables required quantitative measurements based on defined anatomical landmarks:

- Chin deviation.
- Facial thirds.
- Functional chin deviation.
- Maxillary dental midline deviation.
- Profile types according to A.M. Schwarz.
- Nasolabial angle.

The subjective (qualitatively assessable) variables required qualitative visual assessment of facial soft-tissue characteristics:

- Lip competence.
- Mentalis muscle activity.
- Smile line.
- Buccal corridor.
- Supramental fold.
- Lip profile.
- Profile.

The quantitative measurements and the subjective evaluations were both documented directly by the students in a digital evaluation sheet (SoSci Survey GmbH, Munich, Germany). Data entry was conducted through single-choice responses for categorical variables and manual numeric entry for the nasolabial angle.

### Reference standard

Prior to data collection, a reference standard was established through a consensus procedure involving two orthodontic specialists (JS, HKS) and one trainee orthodontist (AB). All photographs and printed facial scans were independently traced and documented using the evaluation sheet. Discrepancies were resolved through discussion until consensus was achieved. This consensus dataset served as the gold standard for subsequent analyses.

### Procedure of the facial soft-tissue analysis

The study was conducted with dental students in their 7th, 9th, and 10th semesters (*n* = 93) as part of the regular orthodontic university curriculum. Participation in the study was voluntary. The students differed in their level of prior experience: 7th-semester students had practiced orthodontic photo analysis for the first time during the semester. 9th-semester students had already completed two semesters of structured training in photo analysis. Shortly before their final examination, 10th-semester students received additional theoretical instruction and were presented with orthodontic patient cases for diagnostic training.

Importantly, none of the semesters included instruction or practical training in tracing printed 3D facial scans because the curriculum exclusively uses 2D images to teach orthodontic soft-tissue analysis.

Prior to the initiation of the study, all participants were provided with standardized instructions on the use of the online evaluation sheet. These instructions contained example images and demonstration entries, which were provided by AB and JS.

The students completed the evaluations on three separate dates, each one week apart, in a lecture hall setting. The following diagnostic modalities were presented in the following order: t1: 2D photographs, t2: 3D color facial scans, t3: 3D grayscale facial scans.

The evaluation process consisted of tracing the printed images using a set square and pen to determine the objective parameters, followed by the assessment of the subjective variables. All measurements and ratings were then entered directly and anonymously into the digital evaluation sheet (SoSci Survey GmbH, Munich, Germany).

For each session, the students received semester-specific participation codes and generated a personal encryption code, which they used consistently across all three time points to ensure anonymous longitudinal assignment.

### Statistical analysis

Statistical analysis was performed using MedCalc^®^ (version 23.1.3; MedCalc Software Ltd, Ostend, Belgium). Only evaluation sheets from students who completed the assessment at the respective time points (t1–t3) were included in the analysis.

For all categorical variables, student responses were compared with a predefined reference standard established by experienced orthodontists. Agreement (correct answer) between student assessments and the reference standard, as well as agreement across different semester cohorts, was quantified using Cohen’s kappa coefficient and reported as percentages of agreement. To enable a systematic evaluation of the direction of disagreement (incorrect answer), ordinal response options were coded numerically according to the order presented in the evaluation sheet (e.g., 1, 2, 3).

## Results

**Fig. 4:**
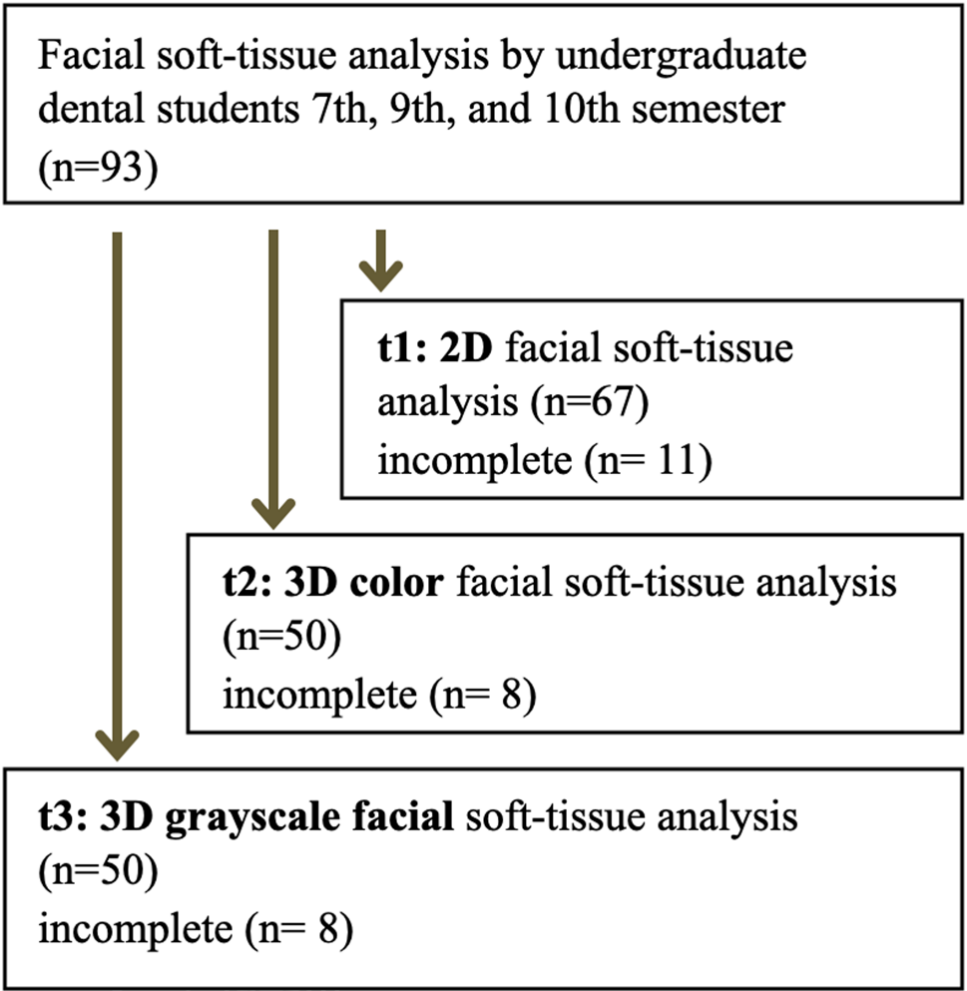
Flowchart of the educational study illustrating student participation, assessment stages, and incomplete datasets across three time points (t1–t3)

At t1, 67 students participated in the assessment of 2D photographs, of whom 11 did not complete the evaluation. One week later (t2), 50 students assessed 3D color images, with 8 incomplete datasets. At t3, conducted one week after t2, 50 students evaluated 3D grayscale images, and 8 students discontinued the assessment (Fig. 4).

### Results of objective variables of soft-tissue analysis

**Fig. 5:**
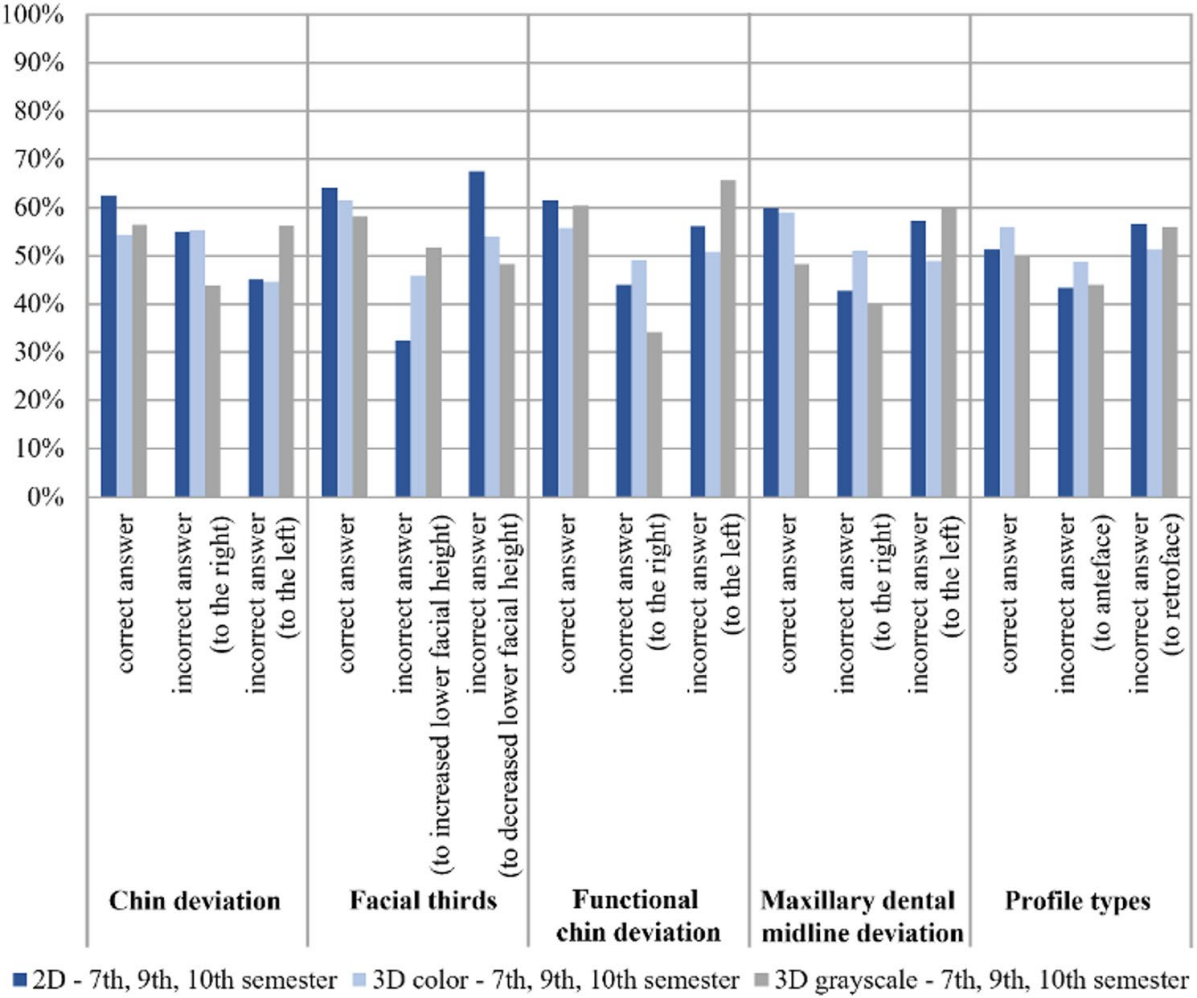
Objective variables of soft tissue analysis: percentage agreement (correct answers) and disagreement (incorrect answers) between the measurements obtained by students in the 7th, 9th, and 10th semesters using 2D, 3D color, and 3D grayscale imaging, compared with the corresponding reference values for each individual variable. Profile types (Profile types according to AM Schwarz)

The objective soft-tissue parameters assessed using 2D, 3D color, and 3D grayscale images were compared with the reference standard for the entire student cohort (Fig. 5).

Overall, agreement was highest for 2D images and lowest for 3D grayscale images across most objective variables. For chin deviation, functional chin deviation, facial thirds, and maxillary dental midline deviation, agreement decreased progressively from 2D to 3D color and further to 3D grayscale images.

An exception was profile classification according to A.M. Schwarz, which demonstrated the highest agreement when assessed on 3D color images compared with both 2D and 3D grayscale images (Fig. 5).

**Fig. 6:**
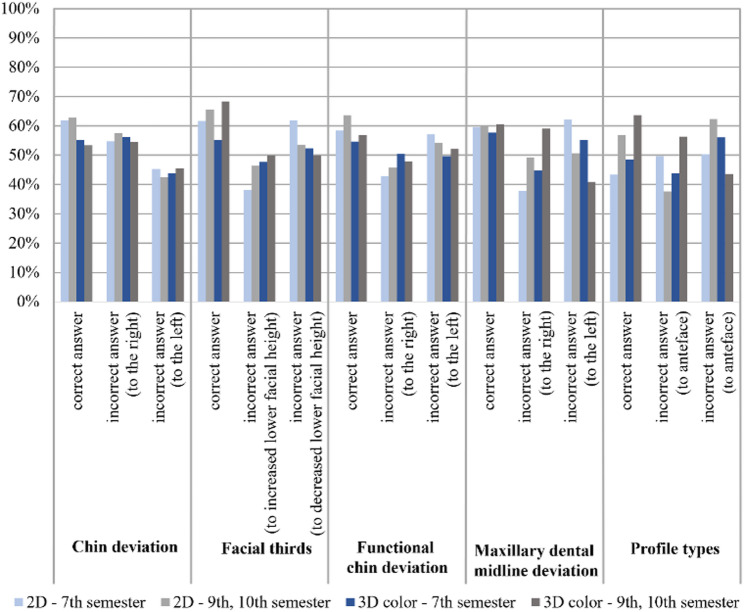
Objective parameters: percentage agreement (correct answers) and disagreement (incorrect answers) between the measurements obtained by students in the 7th, 9th, and 10th semesters using 2D and 3D color imaging, compared with the corresponding reference values for each individual variable. Profile types (Profile types according to AM Schwarz)

Based on the greater clinical relevance of color facial scans, subsequent analyses focusing on the influence of student experience were restricted to comparisons between 2D and 3D color images (see Fig. 6). When stratified by educational level, students in the 9th and 10th semesters showed consistently higher agreement than 7th-semester students for most objective parameters, particularly for facial thirds and profile classification. In contrast, agreement for chin asymmetry under function and maxillary dental midline deviation differed only marginally between semester groups. Within the 7th semester, agreement was generally lower for 3D color images than for 2D photographs, with the exception of profile classification according to A.M. Schwarz. Conversely, students in the higher semesters demonstrated higher agreement in 3D color images for facial thirds, maxillary dental midline deviation, and profile classification (Fig. 6).

### Results of subjective variables of soft-tissue analysis

**Fig. 7:**
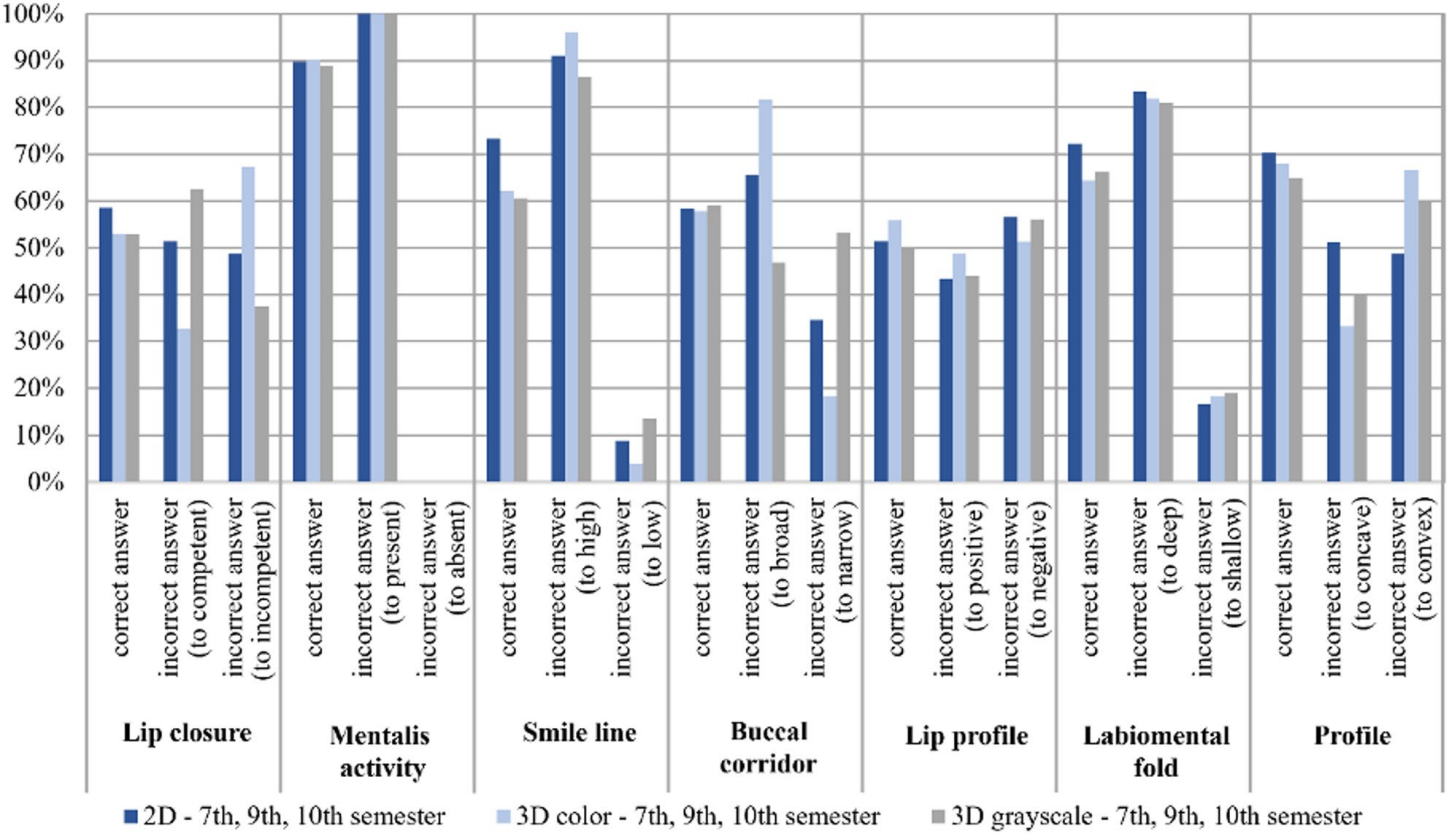
Subjective parameters: percentage agreement (correct answers) and disagreement (incorrect answers) between the measurements obtained by students in the 7th, 9th, and 10th semesters using 2D, 3D color, and 3D grayscale imaging, compared with the corresponding reference values for each individual variable

The subjective soft-tissue parameters assessed using 2D, 3D color, and 3D grayscale images were compared with the reference standard for the entire student cohort (see Fig. 7). Across most subjective variables, overall agreement was highest for 2D photographs and lowest for 3D grayscale images, while 3D color images showed intermediate agreement.

High levels of agreement were observed for mentalis activity across all imaging modalities. For lip closure, agreement was higher in 2D photographs than in scan-derived images. Smile line assessment showed reduced agreement in scan-derived images compared with 2D photographs.

Agreement for buccal corridor width was moderate across modalities, with 3D colors images showing a higher proportion of classifications toward broader categories. For labiomental fold, agreement decreased from 2D photographs to 3D color and further to 3D grayscale images. Profile assessment demonstrated lower agreement for scan-derived images compared with 2D photographs (Fig. 7).

**Fig. 8:**
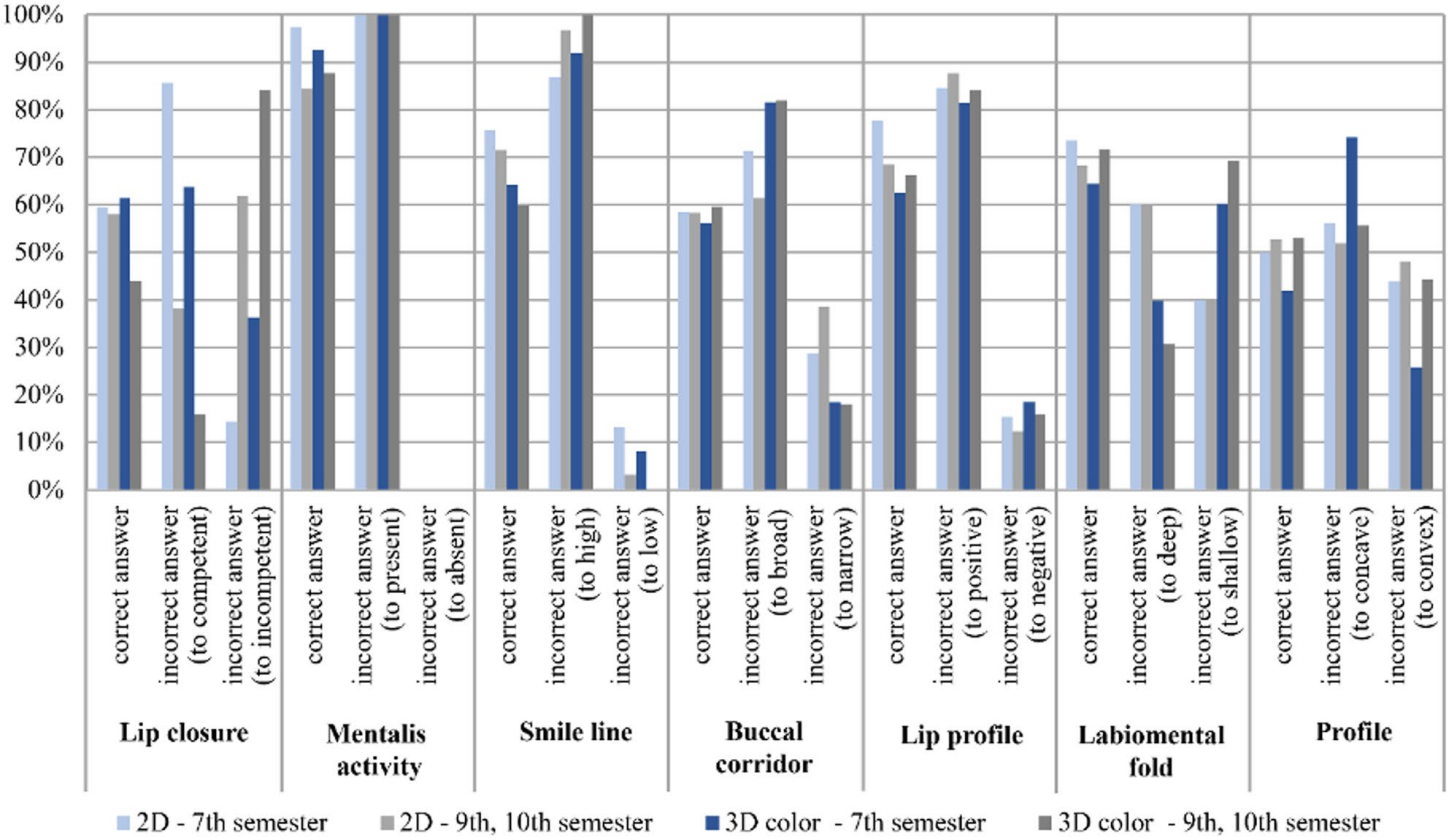
Subjective parameters: percentage agreement (correct answers) and disagreement (incorrect answers) between the measurements obtained by students in the 7th, 9th, and 10th semesters using 2D and 3D color imaging, compared with the corresponding reference values for each individual variable

When stratified by educational level, differences between 7th-semester students and those in the 9th and 10th semesters were generally small for buccal corridor assessment in both 2D and 3D color images (Fig. 8). Lip closure was assessed with higher agreement by 7th-semester students in 3D color images compared with higher semesters, whereas differences in 2D images were minimal. Similarly, 7th-semester students showed higher agreement for mentalis activity and profile in both 2D and 3D color images (Fig. 8).

## Discussion

The present prospective study investigated the transferability of conventional 2D soft-tissue analysis to printed representations of 3D facial scans (color and grayscale) and examined the influence of image modality and undergraduate student experience on diagnostic agreement with a reference standard.

### Objective parameters

Agreement for several objective variables (chin deviation, functional chin deviation, facial thirds, maxillary dental midline deviation, and profile classification according to A. M. Schwarz [10]) was consistently lower for 3D grayscale images than for 2D photographs and, in most cases, also lower than for 3D color images. This finding could be explained by the absence of color surface texture in grayscale scans. As illustrated in Fig. 3, the identification of anatomical landmarks such as the trichion, glabella, pupils, and porion relies on visible surface morphology and color contrast in 2D photographs and color scans. When these color-contrasting features are absent, orientation and landmark identification are impaired, particularly in less experienced observers. Similar effects of surface texture and scan resolution on landmark identification have been reported in previous studies [11–14].

When evaluating the maxillary dental midline deviation, the significantly lower correlation of the 3D grayscale image indicates that the intraoral structures of the central maxillary incisors are more difficult to evaluate. We assume that the reason for this is less likely to be an incorrect drawing of the median sagittal plane and more likely to be an inaccurately represented interdental area of the maxillary central incisors. This appears to have a negative effect on the evaluation of the maxillary dental midline deviation of 3D grayscale. The reduced depiction can be seen in the 3D grayscale facial scan in Fig. 3.

Profile classification according to A. M. Schwarz [10] showed the highest agreement for 3D color images, exceeding that of both 2D and 3D grayscale images. This result supports the interpretation that color scans may enhance the perception of sagittal soft-tissue contours, despite known limitations of stationary scanners in peripheral regions such as the ears. The lower agreement observed for grayscale images is consistent with previously reported reductions in peripheral resolution in stationary facial scanners [15–17].

Although grayscale images are not routinely used in clinical orthodontic practice, they were intentionally included as an experimental condition to isolate the influence of surface texture and color information on landmark identification and diagnostic agreement. By removing texture while preserving 3D morphology, this condition allowed a more differentiated interpretation of how visual cues contribute to soft-tissue assessment. Consequently, the primary clinical implications of the present findings relate to color facial scans rather than to grayscale representations.

### Subjective parameters

For subjective variables, agreement was generally highest for 2D images and lowest for 3D grayscale images, with 3D color images showing intermediate agreement. This pattern is clearly reflected in the results for lip closure, smile line, and profile. The tendency toward a konvex profile and negative lip profile across all image types - particularly pronounced in 3D color images - suggest that enhanced 3D visual information may alter visual perception of orofacial contours rather than improve diagnostic agreement.

The disagreement of the buccal corridor in 3D color images, resulting in frequent classification as broad, may be explained by increased shadowing and contrast in the posterior oral region of color scans (Fig. 2b).

### Influence of scanner technology

The RayFace 200 scanner offers short acquisition times and high patient comfort, which are particularly advantageous in pediatric patients and during smiling recordings, where motion artifacts are a concern (Jarosz et al. 2018; Jindanil et al. 2025). At the same time, the reduced resolution in peripheral areas likely contributed to the lower agreement observed for certain profile-based parameters, especially in grayscale images (Fig. 3c). This trade-off between acquisition speed and spatial resolution is appropriately contextualized within the existing literature [15, 16, 18–22].

### Influence of student experience

The higher level of agreement achieved by students in the 9th and 10th semesters for most objective parameters compared with 7th-semester students can plausibly be attributed to greater clinical experience and repeated exposure to structured training in orthodontic soft-tissue analysis. This effect was observed for both 2D and 3D color images, indicating that diagnostic experience gained with conventional photographs can, at least in part, be transferred to the assessment of color facial scans. These findings are in line with previous reports highlighting the importance of clinical experience and calibration in orthodontic diagnostics [23]. In contrast, several subjective parameters were assessed with higher agreement by 7th-semester students. This finding is likely explained by the close temporal proximity of a dedicated lecture on soft-tissue analysis, which took place approximately one week prior to data collection. In comparison, the most recent corresponding instruction occurred approximately six months earlier for 9th and 10th-semester students. The observed differences underscore the relevance of continuous calibration and regular refresher training.

### Strengths and limitations

A key strength of this study is the standardized, within-subject comparison of imaging modalities: for each patient, corresponding 2D photographs and 3D scan-derived images (color and grayscale) were evaluated using identical criteria and the same assessment form. Further strengths include the use of a predefined expert consensus reference standard and the inclusion of students at different stages of dental education, which enabled a differentiated assessment of the influence of clinical experience on soft-tissue analysis. In addition, classifying disagreement provided clinically and educationally meaningful information beyond overall agreement.

Several limitations should be considered. First, the assessments were conducted sequentially in a fixed order (2D, 3D color, 3D grayscale), which may have introduced order-related effects such as learning, fatigue, or strategy shifts despite the one-week interval between the assessments. Although this sequential structure reflected the progression of the existing curriculum, randomized or crossover designs (e.g., Latin square approaches) would have provided stronger control for potential order effects and should be considered in future studies.

Second, participation differed across time points and incomplete datasets occurred, raising the possibility of attrition bias if students with lower motivation were more likely to discontinue. Specifically, participation decreased from 67 students at t1 to 50 students at t3. As participation in this educational study was voluntary and anonymous, no additional personal or baseline characteristics were collected that would have allowed a formal dropout analysis or an intention-to-treat approach. Consequently, potential differences between students who completed all assessments and those who discontinued cannot be excluded, and some attrition-related selection bias may have occurred.

Third, 3D scans were assessed as standardized printed views rather than as interactive digital 3D datasets; therefore, potentially beneficial features of 3D imaging (rotation, reorientation, zoom, and software-based tools) were not available and may have disadvantaged scan-based assessments.

Furthermore, grayscale scan-derived images represent an experimental condition with limited direct clinical applicability. While their inclusion provided methodological insight into the role of surface texture, conclusions regarding routine orthodontic practice primarily concern color facial scans.

Finally, findings may be influenced by the specific scanner technology. The absence of prior structured training in 3D facial analysis should therefore be considered when interpreting diagnostic agreement across imaging modalities.

The absence of prior structured training in 3D facial analysis should therefore be considered when interpreting diagnostic agreement across imaging modalities. In addition, differences in recent curricular exposure between semesters may have influenced baseline competencies and thereby affected comparability between cohorts. Future course cycles should include standardized preparatory training in both 2D and 3D analysis to ensure more consistent baseline conditions.

Moreover, no formal statistical comparisons between imaging modalities were conducted. Differences in agreement across 2D, 3D color, and 3D grayscale images should therefore be viewed as descriptive trends rather than statistically confirmed differences between modalities. Moreover, no formal statistical comparisons between imaging modalities were conducted. Differences in agreement across 2D, 3D color, and 3D grayscale images should therefore be interpreted as descriptive trends rather than statistically confirmed differences between modalities. In addition, responses were analyzed in predefined categories (e.g., correct vs. incorrect answers), focusing on the direction of change. Given the exploratory educational setting and the relatively small sample size, we considered a descriptive presentation of these trends more appropriate than performing multiple significance tests across all response categories, as this might have increased the risk of overinterpretation. The findings should therefore be interpreted with caution, particularly with regard to generalizability.

### Future directions

Future educational research should explicitly address the methodological limitation of the present study by evaluating fully interactive 3D facial scans within dedicated software environments. In routine clinical and educational workflows, 3D datasets are assessed dynamically, allowing rotation, reorientation, zooming, and multi-angle visualization. These features represent a central advantage of 3D imaging and were intentionally not available in the present standardized printed format.

In this context studies should therefore prioritize the use of interactively visualized 3D datasets in order to simulate authentic clinical application scenarios and to more accurately assess the true diagnostic potential of facial scanning technology in undergraduate education. In particular, implementing structured training modules in digital 3D analysis followed by interactive assessment sessions may provide more realistic insights into how students integrate 3D information into diagnostic reasoning.

Evaluating whether access to true 3D information and targeted calibration improves the accuracy of soft-tissue analysis compared with conventional 2D photographs is of high educational relevance. Moreover, future studies should examine whether these digital approaches facilitate the assessment of complex facial parameters, such as asymmetries and sagittal relationships, and whether they enhance learning outcomes and diagnostic confidence at different stages of undergraduate orthodontic education.

## Conclusion

Conventional 2D orthodontic soft-tissue analysis performed by dental undergraduate students can be transferred to corresponding images derived from facial scans, though with limitations. In particular, the absence of surface texture and the reduced depiction of peripheral and intraoral regions impair diagnostic agreement when using 3D grayscale images. The presence of color surface texture in 3D color scans facilitates landmark identification and improves the assessment of both objective and subjective parameters. Greater clinical experience and repeated training in soft-tissue analysis positively influence diagnostic agreement, especially for objective variables. These findings support the educational integration of color facial scans but also emphasize the need for specific training and appropriate image quality standards.

## Data Availability

The datasets used and analyzed during the current study are available from the corresponding author on reasonable request.

## Abbreviations

2D: two-dimensional
3D: three-dimensional

## References

[1] Arnett GW, Bergman ET. Facial keys to orthodontic diagnosis and treatment planning. Part I. Am J Orthod Dentofac Orthop. 1993;103:299–312. 10.1016/0889-5406(93)70010-L. 10.1016/0889-5406(93)70010-L 8480695

[2] Alqattan M, Djordjevic J, Zhurov AI, Richmond S. Comparison between landmark and surface-based three-dimensional analyses of facial asymmetry in adults. Eur J Orthod. 2015;37:1–12. 10.1093/ejo/cjt075. 24152377 10.1093/ejo/cjt075

[3] Knoops PGM, Beaumont CAA, Borghi A, Rodriguez-Florez N, Breakey RWF, Rodgers W, et al. Comparison of three-dimensional scanner systems for craniomaxillofacial imaging. J Plast Reconstr Aesthet Surg. 2017;70:441–9. 10.1016/j.bjps.2016.12.015. 28161205 10.1016/j.bjps.2016.12.015

[4] Schobben RRP, Rangel FA, Bruggink R, Crins - De Koning MLD, Bronkhorst EM, Ongkosuwito EM. Two experimental methods to integrate intra-oral scans into 3D stereophotogrammetric facial images. Clin Oral Investig. 2025;29:54. 10.1007/s00784-024-06138-8. 10.1007/s00784-024-06138-8 PMC11717827 39786472

[5] Plooij JM, Maal TJJ, Haers P, Borstlap WA, Kuijpers-Jagtman AM, Bergé SJ. Digital three-dimensional image fusion processes for planning and evaluating orthodontics and orthognathic surgery. A systematic review. Int J Oral Maxillofac Surg. 2011;40:341–52. 10.1016/j.ijom.2010.10.013. 21095103 10.1016/j.ijom.2010.10.013

[6] Rangel FA, Maal TJJ, Bergé SJ, Van Vlijmen OJC, Plooij JM, Schutyser F, et al. Integration of digital dental casts in 3-dimensional facial photographs. Am J Orthod Dentofac Orthop. 2008;134:820–6. 10.1016/j.ajodo.2007.11.026. 10.1016/j.ajodo.2007.11.026 19061810

[7] Xiao Z, Liu Z, Gu Y. Integration of digital maxillary dental casts with 3D facial images in orthodontic patients. Angle Orthod. 2020;90:397–404. 10.2319/071619-473.1. 33378431 10.2319/071619-473.1 PMC8032295

[8] Proffit WR. Orthodontic diagnosis: the development of a problem list. Contemp Orthod. 6th edition. 1999;978-0-323-54387-3:147–9.

[9] Faul F, Erdfelder E, Lang A-G, Buchner A. G*Power 3: A flexible statistical power analysis program for the social, behavioral, and biomedical sciences. Behav Res Methods. 2007;39:175–91. 10.3758/BF03193146. 17695343 10.3758/bf03193146

[10] Schwarz AM. Lehrgang der Gebissregelung: Untersuchungsgang (Diagnostik). Urban & Schwarzenberg; 1961.

[11] Ye H, Lv L, Liu Y, Liu Y, Zhou Y. Evaluation of the Accuracy, Reliability, and Reproducibility of Two Different 3D Face-Scanning Systems. Int J Prosthodont. 2016;29:213–8. 10.11607/ijp.4397. 27148978 10.11607/ijp.4397

[12] Maule J, Skelton AE, Franklin A. The Development of Color Perception and Cognition. Annu Rev Psychol. 2023;74:87–111. 10.1146/annurev-psych-032720-040512. 35973406 10.1146/annurev-psych-032720-040512

[13] Thurzo A, Strunga M, Havlínová R, Reháková K, Urban R, Surovková J, et al. Smartphone-Based Facial Scanning as a Viable Tool for Facially Driven Orthodontics? Sensors. 2022;22:7752. 10.3390/s22207752. 36298103 10.3390/s22207752 PMC9607180

[14] Jindanil T, Ponbuddhichai R, Massant C, Xu L, Fontenele RC, Llano-Pérula MCD, et al. Three-Dimensional Facial Imaging: A Comparative Assessment of the Clinical Applicability of State‐of‐the‐Art Technologies for Three‐Dimensional Facial Imaging. Int J Dent. 2025;2025:8822293. 10.1155/ijod/8822293. 40401237 10.1155/ijod/8822293 PMC12094855

[15] Akan B, Akan E, Şahan AO, Kalak M. Evaluation of 3D Face-Scan images obtained by stereophotogrammetry and smartphone camera. Int Orthod. 2021;19:669–78. 10.1016/j.ortho.2021.08.007. 34544662 10.1016/j.ortho.2021.08.007

[16] Lee JD, Nguyen O, Lin Y-C, Luu D, Kim S, Amini A, et al. Facial Scanners in Dentistry: An Overview. Prosthesis. 2022;4:664–78. 10.3390/prosthesis4040053.

[17] Lee GH, Park JH, Park JJ, Lee KC, Lee SM, Moon D. Key considerations for efficient 3-dimensional data integration with a face scanner in the digital era: Product review of the hybrid face scanner. AJO- Clin Companion. 2024;4:351–9. 10.1016/j.xaor.2024.06.004.

[18] Franco De Sá, Gomes C, Libdy MR, Normando D. Scan time, reliability and accuracy of craniofacial measurements using a 3D light scanner. J Oral Biol Craniofac Res. 2019;9:331–5. 10.1016/j.jobcr.2019.07.001. 31388482 10.1016/j.jobcr.2019.07.001 PMC6669702

[19] Antonacci D, Caponio VCA, Troiano G, Pompeo MG, Gianfreda F, Canullo L. Facial scanning technologies in the era of digital workflow: A systematic review and network meta-analysis. J Prosthodont Res. 2022;67:321–36. 10.2186/jpr.JPR_D_22_00107. 36058870 10.2186/jpr.JPR_D_22_00107

[20] Cho R-Y, Byun S-H, Yi S-M, Ahn H-J, Nam Y-S, Park I-Y, et al. Comparative Analysis of Three Facial Scanners for Creating Digital Twins by Focusing on the Difference in Scanning Method. Bioengineering. 2023;10:545. 10.3390/bioengineering10050545. 37237615 10.3390/bioengineering10050545 PMC10215089

[21] Michelinakis G, Apostolakis D, Nikolidakis D, Lapsanis G. Influence of different scan body design features and intraoral scanners on the congruence between scan body meshes and library files: An in vitro study. J Prosthet Dent. 2024;132:454e1. 10.1016/j.prosdent.2024.05.016. 10.1016/j.prosdent.2024.05.016 38879392

[22] Nuytens P, Ruggiero G, Vandeweghe S, D’haese R. Trueness and precision of a handheld, a desktop and a mobile 3D face scanning system: An in vitro study. J Dent. 2025;155:105639. 10.1016/j.jdent.2025.105639. 39978748 10.1016/j.jdent.2025.105639

[23] Alhammadi MS, Halboub E, Fayed MS, Labib A, El-Saaidi C. Global distribution of malocclusion traits: A systematic review. Dent Press J Orthod. 2018;23. 10.1590/2177-6709.23.6.40.e1-10.onl. :40.e1-40.e10. 10.1590/2177-6709.23.6.40.e1-10.onl PMC6340198 30672991

